# Inflammation in Retinal Vein Occlusion

**DOI:** 10.1155/2013/438412

**Published:** 2013-04-03

**Authors:** Avnish Deobhakta, Louis K. Chang

**Affiliations:** Department of Ophthalmology, Columbia University Medical Center, New York, NY 10032, USA

## Abstract

Retinal vein occlusion is a common, vision-threatening vascular disorder. The role of inflammation in the pathogenesis and clinical consequences of retinal vein occlusion is a topic of growing interest. It has long been recognized that systemic inflammatory disorders, such as autoimmune disease, are a significant risk factor for this condition. A number of more recent laboratory and clinical studies have begun to elucidate the role inflammation may play in the molecular pathways responsible for the vision-impairing consequences of retinal vein occlusion, such as macular edema. This improved understanding of the role of inflammation in retinal vein occlusion has allowed the development of new treatments for the disorder, with additional therapeutic targets and strategies to be identified as our understanding of the topic increases.

## 1. Introduction

Retinal vein occlusions (RVOs) are the second most common visually disabling disease affecting the retina, after diabetic retinopathy [1]. Obstruction of retinal venous flow leads to damage of the vasculature, hemorrhage, and tissue ischemia [2]. Occlusions affecting the central retinal vein, or central retinal vein occlusion (CRVO), affect the entire retina, while those affecting lesser tributaries of the venous circulation, the so-called branch retinal vein occlusion (BRVO), affect a portion of the retina. Despite the fact that the disease entity has been known to exist for over 100 years, current treatment options often still leave patients with clinically problematic visual disturbances and overall increased morbidity. RVO generally affects patients in middle age and the elderly population [2], and several studies have identified systemic risk factors, such as hypertension, diabetes, systemic vascular disease, glaucoma, and hypercoagulable states [3, 4]. 

Although proliferative vascular changes can cause significant morbidity (particularly due to subsequent vitreous hemorrhage and neovascular glaucoma), the main reason for decreased visual acuity in both CRVO and BRVO is macular edema [5]. As a result, elucidation of the causes of, as well as treatment for, macular edema has been at the center of large-scale studies on patients with RVO. While the causes for RVO are multifactorial, with local and systemic factors being identified as etiologic, most of the literature generally implicates vascular and inflammatory mediators as being particularly salient [6–8]. Prior to the advent of intravitreal drug delivery, treatment for macular edema for CRVO and BRVO was observation and grid laser photocoagulation, respectively, the latter of which resolved macular edema slowly even under optimal circumstances [9]. The subsequent creation of intravitreal medicines that block vascular endothelial growth factor (VEGF) and the intravitreal delivery of corticosteroids for RVO has led to better clinical outcomes overall [10]. While the focus of much of the literature is currently on the role of anti-VEGF medications in the treatment of RVO, the role of inflammation in both pathogenesis and treatment of RVO is equally exigent. 

## 2. Pathogenesis of Inflammation in RVO

Both systemic and local inflammations have been hypothesized to play a significant role in the etiology of RVO. The predisposing systemic risk factors for RVO include hypertension, diabetes, dyslipidemia, and elevated plasma levels of homocysteine [11–13]. Atherosclerosis, a chronic, low-grade inflammatory condition, has been studied extensively in relation to RVO. Indeed, the systemic risk factors that predispose patients to RVO are also independently associated with atherosclerosis [11, 13]. The initial pathological findings of this condition are composed of monocyte-derived macrophages and T-lymphocytes (purely inflammatory lesions) which later progress to thrombus and clot formation [14]. Results pertaining to the hypothesis of atherosclerosis as a risk factor for RVO have been mixed. Large population-based cross-sectional studies have found that, while the prevalence of RVO is fairly similar across ethnic groups, atherosclerotic disease and markers of inflammation, such as C-reactive protein, were not associated with the disease [15]. In addition, certain genetic polymorphisms that had been previously implicated in atherogenesis, inflammation, and coagulation did not show association with BRVO or CRVO [16, 17]. However, other reports have shown potential links between atherosclerosis (and by extension, systemic inflammation) and RVO. In particular, recent studies have shown that patients with RVO have an increased risk of asymptomatic ipsilateral carotid artery plaques, and those with BRVO often also have decreased aortic distensibility and elasticity, a finding frequently found in patients with atherosclerosis [18, 19]. In addition, pathological studies have shown an atherosclerotic retinal artery at the lamina cribrosa in some patients with CRVO [20]. 

Another mechanism by which systemic inflammation is proposed to lead to RVO is through the induction of systemic hypercoagulability. Many inflammatory chemokines/cytokines are prothrombogenic; for example, interleukin-1 beta, interleukin-6, and tumor necrosis factor Alpha all simultaneously upregulate tissue factor, which is a major activator of the extrinsic coagulation cascade pathway, and downregulate tissue type plasminogen activator, which disrupts fibrinolysis [21–23]. In particular, homocysteine, a plasma element found elevated in patients with chronic inflammatory conditions, such as atherosclerosis, as well as in patients with errors of protein metabolism (homocysteinemia/homocystinuria), can cause adverse systemic thrombotic events. Patients suffering from grossly elevated plasma levels of homocysteine often develop deep vein thromboses, myocardial infarctions, carotid atherosclerosis, and stroke [24]. In a similar fashion to other inflammatory-mediated processes, proposed mechanisms of thrombosis include inhibition of plasminogen activator, inhibition of protein C activation, activation of Factor V, and the inducement of endothelial cell dysfunction [25–27]. Perhaps unsurprisingly, given the strong possible link between hyperhomocysteinemia and hypercoagulation, subsequent case control studies between patients with and without CRVO have demonstrated a robust correlation between CRVO and elevated plasma levels of homocysteine [28, 29]. However, other studies have rightfully pointed out that, given that elevated levels of plasma homocysteine are found in various other chronic inflammatory states, such as atherosclerosis, the association of homocysteinemia with RVO is likely multifactorial [30].

Local inflammation within the eye has also been implicated in the pathogenesis of RVO. In vivo assessment of the vitreous fluid in patients with RVO has demonstrated elevated levels of proinflammatory mediators and lower levels of anti-inflammatory cytokines [31, 32]. In particular, in a major study on inflammatory immune mediators in a group of vitreoretinal diseases, patients with RVO had elevated levels of interleukin-6, interleukin-8, and monocyte chemoattractant protein-1, and patients with CRVO had elevated levels of VEGF, all of which are considered highly proinflammatory [33]. In follow-up studies, patients with macular edema from both BRVO and CRVO were shown to have increased levels of soluble intercellular adhesion molecule-1 (proinflammatory) and decreased levels of pigment epithelium derived factor (anti-inflammatory) [34, 35]. Unsurprisingly, the literature suggests that for larger order vessel disruptions, such as those affecting the central retinal vein or a larger branch retinal vein (“major” BRVO), there are even higher elevations and reductions of the aforementioned pro-inflammatory and anti-inflammatory cytokines, respectively, as compared to smaller branch vessel disruptions [32, 36]. Of particular note is the fact that VEGF is classified as a pro-inflammatory cytokine; while VEGF is famously known for its central role in retinal angiogenesis, recent studies have revealed its role in permitting leukocyte infiltration into the retina—a key initial step in the inflammatory pathway [37, 38].

Macular edema itself has been shown to result from prolonged inflammatory states, such as those seen in uveitis [39]. While the exact mechanism for how inflammation actually causes macular edema is still unclear, the prevailing theory includes the instigation of pro-inflammatory cytokines that subsequently damage retinal cells, particularly retinal pigment epithelial cells, which leads to fluid leakage into the retina [15]. In addition, the retinal ischemia seen with RVO has also been postulated to lead to a pro-inflammatory milieu, with the added insult of increased vascular permeability partially due to a breakdown of the blood-retinal barrier [40]. Given these conditions, treatment options for RVO preventing inflammation were developed.

## 3. Treatment of Inflammation in RVO

 While the mainstay of treatment for systemic inflammatory states has been oral or intravenous corticosteroids, this method of administration precluded their effective use for ocular conditions given the side effect profile of long-term steroid use. In addition, topical steroids do not penetrate the posterior segment of the eye in an efficacious manner [5]. However, injecting corticosteroids directly into the vitreous cavity allows for a targeted, high dose use of the medications for ocular inflammatory conditions with a low side effect profile. Currently, the major anti-inflammatory medications in use for the treatment of RVO are intravitreal triamcinolone acetonide (IVTA) and the newly developed dexamethasone intravitreal implant.

 Triamcinolone acetonide is a synthetic glucocorticoid that has a potency that is five times that of cortisol and has been reported to remain in the eye for months to years after its initial injection [41, 42]. Initial use of IVTA for treatment of CRVO resulted in significantly improved anatomical changes within the macula [8, 43, 44]. As a result, the SCORE (Standard Care versus Corticosteroid for Retinal Vein Occlusion) trial was launched by the National Eye Institute. The study consisted of two multicenter, randomized controlled clinical trials comparing the efficacy of IVTA versus standard of care for both BRVO and CRVO [45, 46]. The SCORE-BRVO arm placed patients in cohort groups which received 1 mg of IVTA, 4 mg of IVTA, or standard of care (macular grid laser photocoagulation). The results demonstrated no difference between the three groups in terms of visual outcome; however, there was an increased incidence of adverse side effects such as glaucoma, cataract, and injection-related problems in the IVTA groups relative to the laser group [46]. Expectantly, the adverse side effects were more pronounced in patients receiving the higher dosage of IVTA. As a result, the study concluded that for BRVO, macular grid laser photocoagulation should remain the gold standard for treatment. The SCORE-CRVO arm placed patients in cohorts similar to the SCORE-BRVO arm; however, the results demonstrated that both IVTA groups were superior to observation (standard of care for CRVO) in both visual acuity and anatomic resolution of macular edema [45]. These beneficial changes occurred as early as 4 months into treatment and persisted for 24 months. The study also demonstrated a reduced incidence of adverse side effects in the 1 mg IVTA group; as a result, this dosage has been preferred by some in the treatment of CRVO.

 Given the partial success of temporary intravitreal corticosteroids, a method of delivering corticosteroids in a manner that obviated the need for multiple injections was developed. The dexamethasone implant is a biodegradable copolymer of both lactic and glycolic acids with micronized dexamethasone that gradually releases the dose of the steroid over a period of months via the pars plana [5]. The GENEVA trials were two phase III trials that tested the effect of dexamethasone implants (in the 0.35 mg and 0.7 mg dosages) versus sham injections in patients with BRVO and CRVO [47, 48]. The results for the BRVO study group were mixed; while there was a trend towards better visual acuity in the dexamethasone implant groups after 6 months, there was a statistically significant improvement of acuity in the dexamethasone implant groups after 3 months. A similar finding, though less in magnitude, was seen in the CRVO group. Patients tolerated the implant well, with a minority of patients developing medically manageable glaucoma and cataract [47]. Given the results of the GENEVA trials, some advocate use of the implant for patients with a relatively short duration of macular edema [48]. Others have suggested that the dexamethasone implant may be useful for less frequent occurrences of macular edema secondary to RVO, such as those occurring in postvitrectomized eyes with CRVO, and those with long-standing BRVO and chronic edema [49, 50].

However, considering that the pathogenesis of inflammation in RVO also includes VEGF as a key mediating cytokine, the advent of intravitreal anti-VEGF medications and their role in the treatment of RVO are especially salient. Ranibizumab is a monoclonal, humanized antibody fragment that binds to all VEGF isomers. Two randomized controlled trials were established to determine the efficacy and safety of ranibizumab in the treatment of RVO: BRAVO (BRVO) and CRUISE (CRVO) [51, 52]. In both BRAVO and CRUISE studies, patients with fovea involving macular edema within the prior 12 months were given monthly ranibizumab injections of either 0.3 mg, 0.5 mg, or sham injections. In the BRAVO study, patients who were not responding to treatment were eligible to receive rescue laser photocoagulation (standard of care) after 3 months. At 6 months of treatment, patients in the ranibizumab groups in both studies had significantly higher average gains in visual acuity, significantly higher proportions of patients gaining at least 15 letters of vision, and significantly lower mean foveal thicknesses relative to the sham injection group. In addition, patients maintained this vision with continued injections through 12 months; intriguingly, patients in the sham group who were subsequently given ranibizumab injections after the 6-month period enjoyed beneficial visual and anatomic changes—however, their final visual acuities were generally less than those in the ranibizumab groups, engendering a discussion on whether there was a visual penalty resulting from a delay in treatment [53, 54]. Similarly beneficial effects in smaller studies have been noted with another anti-VEGF antibody, bevacizumab; however, many of the studies also mention a high recurrence rate and relatively short-term-efficacy [55–60].

Given the beneficial treatment outcomes of both intravitreal steroid and intravitreal anti-VEGF medications, a few reports have attempted to ascertain whether a synergistic effect might exist. One study found no significant difference in outcome between patients with CRVO who only received bevacizumab versus patients who received both bevacizumab and triamcinolone [61]. Another study attempted to assess whether patients with RVO who received both bevacizumab and a dexamethasone implant (0.7 mg) had significantly better outcomes than those who received only the dexamethasone implant [62]. The patients in the combination group were given the dexamethasone implant 2 weeks after the first injection of bevacizumab. Most patients (65 percent) were being treated for BRVO. The primary outcome was the time required for reinjection based on existing OCT and visual data. While most patients gained vision, a small minority did not require a retreatment with an additional bevacizumab injection during the 6-month study. While the data suggests that there may be a synergy between anti-VEGF medications and steroids, further study is required.

## 4. Conclusion

RVO is a highly prevalent cause of vision loss in the world. While the causes for RVO are multifactorial, both local and systemic inflammations have been found to be highly contributory factors. Along with photocoagulation, medications that reduce the level of inflammation in the eye, specifically triamcinolone and the dexamethasone implant, have been shown to provide beneficial results for patients with certain forms of RVO. Coupled with the explosion of anti-VEGF medications, such as ranibizumab and bevacizumab, the treatment of RVO is destined to change. Further study of the role of inflammation in the pathogenesis and propagation of RVO will aid in the identification of therapeutic targets and the development of new treatment modalities for this disease.
